# CRISPR/Cas9-mediated uORF engineering enhances tanshinone biosynthesis in *Salvia miltiorrhiza*

**DOI:** 10.1093/hr/uhaf249

**Published:** 2025-09-17

**Authors:** Jin Shao, Bowen Peng, Han Zheng, Ling Li, Dalu Li, Xinyi Hu, Luqi Huang, Kexuan Tang

**Affiliations:** Frontiers Science Center for Transformative Molecules, Joint International Research Laboratory of Metabolic and Developmental Sciences, Plant Biotechnology Research Center, Fudan-SJTU-Nottingham Plant Biotechnology R&D Center, School of Agriculture and Biology, Shanghai Jiao Tong University, Shanghai 200240, China; Frontiers Science Center for Transformative Molecules, Joint International Research Laboratory of Metabolic and Developmental Sciences, Plant Biotechnology Research Center, Fudan-SJTU-Nottingham Plant Biotechnology R&D Center, School of Agriculture and Biology, Shanghai Jiao Tong University, Shanghai 200240, China; State Key Laboratory of Dao-di Herbs, National Resource Center for Chinese Materia Medica, China Academy of Chinese Medical Sciences, Beijing 100700, China; Frontiers Science Center for Transformative Molecules, Joint International Research Laboratory of Metabolic and Developmental Sciences, Plant Biotechnology Research Center, Fudan-SJTU-Nottingham Plant Biotechnology R&D Center, School of Agriculture and Biology, Shanghai Jiao Tong University, Shanghai 200240, China; Shanghai Agricultural Technology Extension Service Center, Shanghai 201103, China; Frontiers Science Center for Transformative Molecules, Joint International Research Laboratory of Metabolic and Developmental Sciences, Plant Biotechnology Research Center, Fudan-SJTU-Nottingham Plant Biotechnology R&D Center, School of Agriculture and Biology, Shanghai Jiao Tong University, Shanghai 200240, China; State Key Laboratory of Dao-di Herbs, National Resource Center for Chinese Materia Medica, China Academy of Chinese Medical Sciences, Beijing 100700, China; Frontiers Science Center for Transformative Molecules, Joint International Research Laboratory of Metabolic and Developmental Sciences, Plant Biotechnology Research Center, Fudan-SJTU-Nottingham Plant Biotechnology R&D Center, School of Agriculture and Biology, Shanghai Jiao Tong University, Shanghai 200240, China; Yazhouwan National Laboratory, Sanya, Hainan 572024, China

## Abstract

Tanshinone accumulation serves as a critical determinant of medicinal value in *Salvia miltiorrhiza* cultivars. Precise fine-tuning of tanshinone biosynthesis while preserving elite agronomic traits remains a pivotal challenge in molecular breeding. Here, we report, for the first time, the successful application of CRISPR/Cas9-mediated upstream open reading frame (uORF) editing in medicinal plants to enhance the production of specialized metabolites. Five evolutionarily conserved uORFs identified in the 5′ leader sequence of the key diterpene synthase gene *SmCPS1* were strategically edited to modulate post-transcriptional regulation. Homozygous mutants engineered through precision gene editing exhibited 1.19- to 1.81-fold enhanced tanshinone accumulation compared to the controls, correlating with coordinated transcriptional activation of core biosynthetic genes (*SmHMGR1*, *SmKSL1*, *SmCYP76AH1*, *SmCYP76AH3*). Integrative molecular analyses demonstrated unchanged *SmCPS1* transcript levels and enhanced protein accumulation, mechanistically confirming uORF-mediated translational potentiation of the cognate main ORF. This study establishes uORF engineering as a robust platform for predictable metabolic engineering in *S. miltiorrhiza* plants. Future applications could expand this strategy to uORFs of rate-limiting enzymes or transcriptional regulators, enabling multidimensional optimization of high-value metabolites in medicinal species.

## Introduction

*Salvia miltiorrhiza* Bunge (Danshen), a Lamiaceae family perennial herb with millennium-long medicinal applications in traditional Chinese medicine (TCM), was first pharmacologically documented in the Shennong Bencao Jing (Divine Farmer’s Materia Medica) [1]. The official medicinal material derives from the dried roots and rhizomes of this species, characterized by its abbreviated growth cycle, diploid genome, facultative outcrossing reproductive strategy, and various reproductive modes, making it an ideal model organism for TCM modernization research [2]. The phytochemical diversity of *S. miltiorrhiza* underpins its multifaceted pharmacological profile. Extensive research has demonstrated its therapeutic efficacy in managing cardiocerebrovascular disorders, with additional applications in combating malignant tumors, liver cirrhosis, and diabetes mellitus through mechanisms such as blood circulation enhancement and direct cytoprotective actions [3]. The plant's bioactive constituents comprise two principal classes: (i) water-soluble phenolic acids demonstrating marked antioxidant capacity; and (ii) lipid-soluble diterpenoids exhibiting potent pharmacological activity [4]. Notably, the periderm tissue of the plant’s characteristic red roots serves as the primary biosynthetic site for tanshinones, a clinically significant class of abietane-type norditerpenoid quinones. Phytochemical investigations have identified over 40 structurally distinct tanshinones and related derivatives, including but not limited to: Tanshinone I (Tan I), Tanshinone IIA (Tan IIA), Cryptotanshinone (CPT), Dihydrotanshinone (DHT) [5].

Tanshinones are synthesized through sequential catalytic steps utilizing the terpenoid precursors isopentenyl pyrophosphate (IPP) and dimethylallyl diphosphate (DMAPP). The biosynthesis occurs in the following stages: (i) precursor synthesis via the mevalonic acid (MVA) pathway and the 2-C-methyl-d-erythritol 4-phosphate (MEP) pathway, which generates universal isoprenoid units. (ii) Carbon skeleton assembly mediated by diterpenoid synthase, forming the core scaffold of tanshinones. (iii) Structural diversification is driven by CYP450 enzymes, which catalyze oxidative post-modifications to produce functionally complex tanshinone derivatives [6–8]. In the terpenoid biosynthetic pathway, geranylgeranyl diphosphate (GGPP) undergoes initial cyclization via SmCPS1 to form normal copalyl diphosphate. This enzymatic product is subsequently converted by SmKSL1 through catalytic cyclization and structural rearrangement, yielding miltiradiene, the essential biosynthetic precursor for tanshinone formation. Among the characterized *SmCPS1–5* genes, only *SmCPS1* was demonstrated to have the highest catalytic activity in root tanshinone biosynthesis, correlating with its predominant transcript levels [6]. *SmCPS1* homozygous mutant hairy root cultures exhibited complete depletion of tanshinones (CPT, Tan IIA, and Tan I), whereas phenolic acid metabolite profiles remained unaltered [9]. Many transcription factors, including *SmMYB97*, *SmERF6/SmERF73*, and *SmbHLH148*, positively regulate tanshinone biosynthesis through direct binding to the *SmCPS1* promoter [10–13]. These findings collectively establish *SmCPS1* as a key regulatory hub in tanshinone biosynthesis, whose strategic manipulation through metabolic engineering demonstrates significant potential for targeted enhancement of pharmaceutically important diterpenoids in *S. miltiorrhiza*.

Gene expression is precisely regulated through a tripartite regulatory cascade encompassing transcriptional initiation, translational modulation, and posttranslational modifications [14]. The frequent discordance between cellular protein levels and their corresponding mRNA abundance highlights the pivotal role of translational control mechanisms in shaping proteome composition. This regulatory layer is a critical node in modulating protein synthesis efficiency independent of transcript availability [15, 16]. Recent transcriptome-wide analyses have demonstrated that eukaryotic 5′ untranslated regions (UTRs) commonly contain uORFs. These evolutionarily conserved cis-regulatory elements typically function as repressors of downstream main ORF (mORF) translation through a pioneering ribosome scanning mechanism [17–19]. The preferential engagement of ribosomes with uORFs establishes a kinetic competition that substantially impedes translation initiation at the principal coding sequence, thereby enabling dynamic regulation of protein synthesis in response to cellular stimuli [15]. According to the position of the stop codon, uORFs are divided into three types in eukaryotes. In Type 1 (Nonoverlapping), the stop codon of uORFs is located upstream of the ATG initiation codon of mORFs, and approximately 85% of known uORFs belong to this type. In Type 2 (out-of-frame overlapping), the stop codon of uORFs lies in the mORF coding region; In Type 3 (N-terminal extensions), the initiation codon of uORFs is upstream of mORFs, but both are in the same reading frame and share the same stop codon [20]. Cross-species comparative studies demonstrate remarkable evolutionary conservation of this regulatory architecture, with bioinformatic annotations revealing uORF prevalence in 49% of human transcripts, 44% of murine transcripts, and 37% of *Arabidopsis thaliana* mRNA molecules [21]. Notably, genome editing approaches targeting uORFs have been effectively implemented across plant species to optimize agronomic performance through coordinated modulation of developmental processes and stress adaptation networks. Targeted disruption of inhibitory uORFs generates nontransgenic crop variants with agronomically superior phenotypes [22, 23]. Furthermore, uORF-mediated translational control enables the stimulus-responsive expression of mORFs, dynamically coupling protein synthesis to cellular cues such as ionic/metabolic homeostasis, phytohormone fluctuations, environmental signals, and immune activation [24]. This posttranscriptional regulatory paradigm achieves spatiotemporal precision in tuning mORF translation rates, ensuring context-appropriate protein stoichiometry under fluctuating physiological demands. Naturally occurring polymorphisms within the *GmPHF1* uORF region modulate GmPHF1 protein abundance through tissue-specific regulatory mechanisms, thereby driving natural variation in phosphorus uptake efficiency across soybean germplasm [25]. The modified uORF version of rice glutamine synthetase 2 (*OsGS2*) enhances the translational repression of *OsGS2*, providing extensive plant defense and growth adaptation benefits [26]. Genomic editing of *LsGGP2* uORF removes its translation inhibition of a crucial enzyme in vitamin C biosynthesis, enabling lettuce plants to endure oxidative stress and generate a surplus of antioxidant metabolites [27]. Modification of the *OsDLT* 5′UTR enabled the generation of rice plants exhibiting variations in plant height and tiller number [28]. However, there has been no report on the regulation of valuable pharmaceutical compounds by uORF engineering in the medicinal species. The potential of uORF-based genetic engineering to enhance bioactive compound biosynthesis remains unexplored in medicinal species.

In this work, CRISPR/Cas9-mediated genome editing of the *SmCPS1* uORF domain was implemented to fine-tune translational efficiency and amplify tanshinone biosynthesis in *S. miltiorrhiza*. These results delineate post-transcriptional regulatory mechanisms governing diterpenoid metabolism in *S. miltiorrhiza*, thereby establishing a uORF-targeted molecular breeding paradigm for engineering elite germplasm with hyperaccumulation of pharmaceutically active tanshinones.

## Results

### Multiplexed CRISPR-Cas9 targeting for uORF knockout in *SmCPS1*

**Figure 1:**
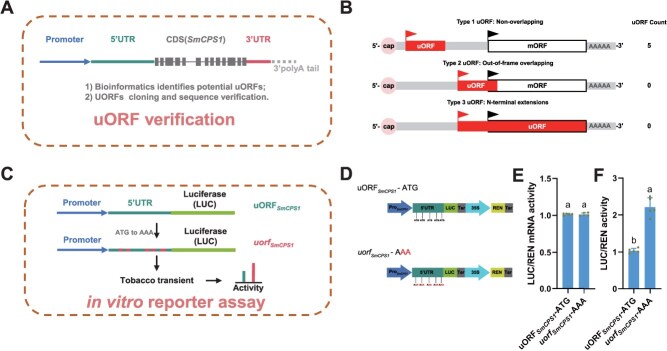
*SmCPS1* uORF verification and in vitro reporter assay. (A) The schematic illustration of the uORF verification in the *SmCPS1* gene. (B) Classification type and count of uORFs in the *SmCPS1* 5′ leader sequence. (C) The schematic diagram of the *SmCPS1* uORF *in vitro* reporter assay is presented. (D) Validation of the *SmCPS1* uORF vector construction using the Dual-LUC reporter system. All uATG initiation codons of *SmCPS1,* five uORFs were systematically mutated to AAA sequences through site-directed mutagenesis. E and F, LUC/REN mRNA activity, and LUC/REN activity. The bars show the mean ± SD (*n* = 5).

**Figure 2:**
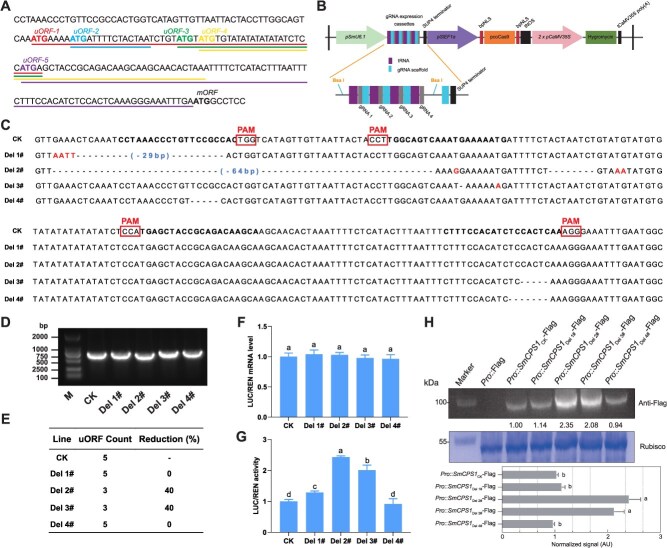
Acquisition of *SmCPS1* uORF mutants and the impact of uORF on the transcriptional and translational levels. (A) The nucleotide architecture of uORFs within the 5′UTR of wild-type *SmCPS1* was systematically analyzed. Initiation codons (ATG), including both the upstream initiation site (uATG) and main initiation codon (mATG), are highlighted in bold, while distinct uORF sequences are annotated with underlining for visual discrimination. (B) Schematic representation of the editing vector targeting the uORF region of the *SmCPS1* gene. (C) Comparative nucleotide alignment schematics of CK versus edited alleles (Del 1#–Del 4#). Protospacer adjacent motifs (PAMs) are highlighted in boxes. (D) Agarose gel electrophoretogram of uORF amplicons from CK and edited lines. (E) Quantitative analysis of uORF prevalence and relative depletion percentages versus CK controls. (F) Transcriptional profiling via uORF-driven Dual-LUC assays comparing CK and mutant genotypes. (G) Translational activity assessment employing uORF-driven Dual-LUC reporters in control and edit lines. (H) Analysis of SmCPS1 protein abundance with edited uORF types in *N. benthamiana*. The *Pro_SmCPS1_*-uORF_(CK, Del 1#–Del 4#)_:: *SmCPS1*–3 × Flag construct was transiently co-transformed into *N. benthamiana* leaves via *Agrobacterium*-mediated transformation. Western blotting was performed using an anti-Flag antibody. The Coomassie blue staining of the RuBisCO large subunit gel demonstrated equivalent sample loading. Values indicate the relative intensity of SmCPS1 protein bands normalized using ImageJ software. AU (Arbitrary Units). The results were reproducible in three independent experiments, with similar outcomes. The values are from a single experiment. Error bars represent ±SD from triplicate biological replicates (*n* = 3). Statistical comparisons used *t*-tests (GraphPad Prism v8.0).

In purple-flowered *S. miltiorrhiza*, the *SmCPS1* gene exhibits predominant root-specific expression, with substantial transcript accumulation also detected in stems and flower tissues. qRT-PCR quantification revealed markedly reduced expression levels in leaf tissues compared to floral tissues, exhibiting a reduction to less than 2% of floral expression levels (Fig. S1). In this study, for targeting the critical regulatory role of *SmCPS1* in the tanshinone biosynthesis pathway, we developed a genome editing strategy focusing on the uORF elements within the 5′UTR of *SmCPS1* (Fig. 1A). Integrated bioinformatics analysis combining genomic and transcriptomic databases identified 5 uORFs (designated uORF1–5) within the 5′UTR region of *SmCPS1*. All identified uORFs were classified as Type 1 (uORF count, *n* = 5), exhibiting lengths of 57, 15, 30, 54, and 84 base pairs, respectively (Figs 1B, 2A, and Fig. S2). Functional validation using a dual-luciferase reporter system demonstrated that mutation of all five uATG initiation codons (ATG → AAA) in *uorf_SmCPS1_* under its native *SmCPS1* promoter significantly enhanced the LUC/REN activity ratio (2.214 vs 1.035 in wild type) without altering transcriptional levels (Fig. 1C–F). The introduced modifications in the *SmCPS1* uORF primarily enhance translational efficiency, not transcriptional regulation, during heterologous expression. The specific endogenous effects of these sites warrant further investigation.

Given the *SmCPS1* 5′UTR architecture containing abundant uATG motifs and the restricted availability of CRISPR/Cas9-compatible PAM sites, we strategically designed four tRNA-linked sgRNAs targeting proximal uATG regions to generate plants with diverse editing profiles (Fig. 2B, Table S1). Using EHA105 *Agrobacterium*-mediated transformation with *SmCPS1* uORF genome editing vector, we achieved 32.26% transformation efficiency (30 transgenic events/93 explants) and 83.33% editing efficiency (25 edited lines/30 transgenic events), with diverse editing patterns: 43.33% heterozygous (13/30), 20% chimeric (6/30), and 20% homozygous (6/30) (Figs S3 and S4A). Four uORF-edited lines (Del 1#–Del 4#) with distinct knockout patterns were selected from six homozygous mutants for functional validation of *SmCPS1* uORF regulatory mechanisms (Fig. 2D). Sanger sequencing was employed to confirm distinct knockout editing details in all four homozygous mutants. Line Del 1# exhibited a 29-bp deletion with a 4-bp insertion in the coding sequence, preserving all five uATG initiation codons. Del 2# displayed two frameshift deletions (64 and 8 bp) accompanied by single- and double-nucleotide substitutions, resulting in the disruption of two critical uATG motifs. The Del 3# lineage manifested a combinatorial mutation profile comprising a 5 bp deletion, single-nucleotide deletion, and base substitution, similarly eliminating two uATGs. Del 4# line presented 5 and 7 bp deletions in the 5′UTR's proximal and distal regions, respectively, with no translational initiation codon impairments observed (Fig. 2B, C and Fig. S5). Intriguingly, while structural modifications were introduced in the Del 1# line, both uORF quantity (5 uORFs) and type remained identical to CK (controls, lines from the same tissue culture background without CRISPR targeting). This conservation creates a unique experimental system for investigating how 5′-proximal nucleotide deletions upstream of uORF sequences influence *SmCPS1* regulation. Quantitative analysis of uORF populations showed significant reductions in Del 2# and Del 3# (3 uORFs each, 40% decrease) and Del 4# (5 uORFs) compared to CK (Fig. 2E, S7). Del 2# and Del 3# displayed 40% and 60% reductions in Type 1 uORFs, respectively, with Del 3# showing concomitant acquisition of a Type 3 uORF. Notably, the Del 4# line maintained CK uORF count but demonstrated a single reduction in Type 1 uORFs accompanied by an additional Type 2 uORF count (Fig. S4B). The differential impacts of these four distinct uORF sequences on *LUC* gene expression were assessed through transient dual-luciferase assays in tobacco, with comparative analysis conducted at both transcriptional and translational levels. The results demonstrated that none of the four mutations induced significant alterations in the transcriptional level of the transiently expressed *LUC* gene. It is worth noting that the relative translational efficiency ratio of the *LUC* gene exhibited a descending order: Del 2# > Del 3# > Del 1# > Del 4#. The Del 4# line showed comparable translational efficiency to the wild-type uORF-containing sequence (Fig. 2F and G). Due to the lack of an antibody specific for the target *SmCPS1* protein, we expressed *SmCPS1* tagged with a Flag epitope heterologously. Western blot analysis revealed that the deletion of uATG in Del 2# and Del 3# resulted in varying degrees of elevated SmCPS1 protein expression. Additionally, the SmCPS1 protein levels in Del 1# and Del 4#, where the uORF number remained unaltered, showed no significant difference compared to the CK (Fig. 2H).

### The uORF editing enhances tanshinone biosynthesis

To investigate the effects of uORFs on *SmCPS1* expression in the *S. miltiorrhiza* plant, we performed qRT-PCR analyses of CK and *SmCPS1* uORF knockout lines. The results demonstrated no significant differences in *SmCPS1* mRNA levels between four uORF mutants and CK plants in both roots and leaves (Fig. S6). Combined with transient expression assays in tobacco and stable qRT-PCR analyses of gene transcription in *S. miltiorrhiza* plants, we can conclude that uORF mutations in *SmCPS1* enhance translational efficiency rather than elevating its transcriptional activity.

**Figure 3:**
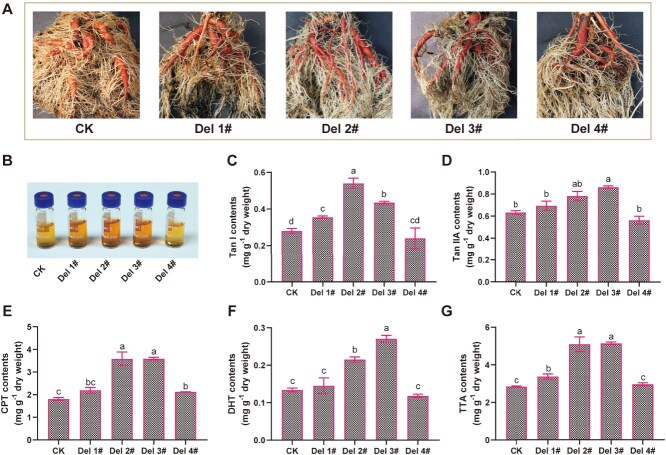
Root phenotypic characterization and tanshinone quantification. (A) The phenotypes of the roots of the CK and uORF knockout lines. (B) Comparison of the color of tanshinone extracts of CK and uORF knockout lines (Del 1#–Del 4#). (C–G) Tanshinone contents were measured in transgenic roots of CK and uORF-knockout lines. Tan I (Tanshinone I), Tan IIA (Tanshinone IIA), CPT (Cryptotanshinone), DHT (Dihydrotanshinone), and TTA (total tanshinone accumulation).

Phenotypic analysis of uORF-edited mutants revealed distinct tanshinone accumulation patterns in root systems, with Del 1#, Del 2#, and Del 3# lines exhibiting characteristic darker red pigmentation compared to wild-type controls, while Del 4# maintained baseline coloration. Corresponding chromatic profiles in methanol extracts corroborated these visual phenotypes (Fig. 3A and B). To investigate the metabolic regulatory effects of *SmCPS1* uORFs mutations, we quantified tanshinone content in mature roots of one-year-old *S. miltiorrhiza* plants using UHPLC-TQMS. Compared to CK controls, the Del 1# line exhibited 26.7% increases in Tan I contents, respectively, whereas CPT, Tan IIA, and DHT showed no significant enhancement. The Del 2# line displayed marked increases in Tan I (92.9%), Tan IIA (23.4%), CPT (98.2%), and DHT (60.5%). Similarly, Del 3# line demonstrated elevated levels of Tan I (55.4%), Tan IIA (36.3%), CPT (98.8%), and DHT (102.7%). In contrast, the Del 4# line maintained Tan I, Tan IIA, and DHT levels comparable to CK, with only CPT contents showing a 17.7% increase (Fig. 3C–F). Comparative analysis of total tanshinone accumulation (TTA) revealed substantial enhancements in *SmCPS1* uORF-edited mutants Del 2# and Del 3#, with TTA increasing from 2.85 mg/g dry weight (DW) in CK controls to 5.11 mg/g DW (1.79-fold) and 5.15 mg/g DW (1.81-fold), respectively. Del 1# exhibited moderate TTA elevation (3.39 mg/g DW; 1.19× wild-type levels), whereas Del 4# maintained baseline tanshinone production indistinguishable from unmodified plants (Fig. 3G). These results collectively demonstrate differential impacts of uORF-edited variants on tanshinone biosynthesis, with Del 2# and Del 3# exhibiting the most pronounced accumulation effects.

### *SmCPS1* uORF mutation regulates the tanshinone biosynthesis genes

**Figure 4:**
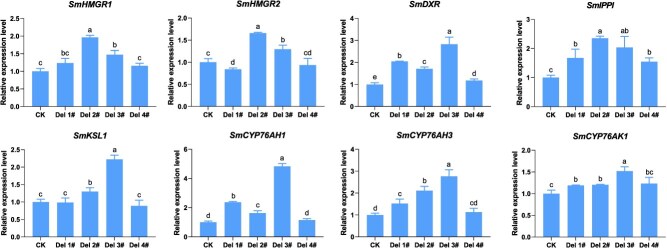
Expression levels of the key enzyme genes of the tanshinone synthesis branch. Abbreviation: *HMGR*, 3-hydroxy-3-methylglutaryl-CoA reductase; *DXR*, 1-deoxy-d-xylulose 5-phosphate reductoisomerase; *IPPI*, isopentenyl diphosphate isomerase; *CYP*, Cytochrome P450-dependent monooxygenases. Vertical bars indicate standard deviations (SD) derived from three independent biological replicates. Statistical analyses were performed using unpaired Student's *t* tests in GraphPad Prism 8 (v.8.0), with different superscript letters denoting significant differences (*P* < 0.05).

To elucidate the regulatory mechanism underlying *SmCPS1* uORF-mediated tanshinone accumulation, a comparative qRT-PCR analysis of pathway-associated genes was conducted. Intriguingly, CRISPR-edited *SmCPS1* uORF knockout lines exhibited coordinated regulation of mid-/downstream tanshinone biosynthetic node pathways. In the MVA pathway, qRT-PCR analysis revealed 1.96- and 1.47-fold increases in *SmHMGR1* and *SmHMGR2* relative expression levels in Del 2# and Del 3# compared to the CK controls, whereas Del 1# and Del 4# showed no significant alterations. Transcript levels of *SmHMGS* and *SmHMGR3* remained unchanged across all Del 1# to Del 4# mutants. In the MEP pathway, *SmDXS2* and *SmDXS3* maintained stable transcription profiles in all four uORF-edited lines, while *SmDXR* expression had a 0.71- to 1.83-fold increase relative to CK plants. Expression levels of *SmIPPI* were moderately increased (1.54- to 2.35-fold) across mutant lines, with Del 2# and Del 3# exhibiting the most pronounced upregulation (Fig. 4). In all four *SmCPS1* uORF mutant lines, isopentenyl pyrophosphate synthase genes (e.g. *SmGGPPS*) showed no significant alterations, whereas the diterpene synthase *SmKSL1* exhibited 1.30- and 2.22-fold elevated expression in Del 2# and Del 3# compared to wild-type controls (Fig. 4 and Fig. S8). Cytochrome P450 enzymes associated with miltiradiene branching (*SmCYP76AH1* and *SmCYP76AH3*) displayed peak inductions of 4.83- and 2.76-fold in Del-2# and Del-3# mutant lines, respectively, while *SmCYP76AK1* expression remained unaffected. Concomitant analysis revealed a modest 1.27 to 1.36-fold increase in *SmTIIAS* transcript levels (Fig. 4 and Fig. S8).

## Discussion

Tanshinones, bioactive diterpenoids enriched in *S. miltiorrhiza* root tissues, are demonstrated to have clinically validated efficacy in managing cardiovascular pathologies, driving surging pharmaceutical demand. This escalating market imperative necessitates the strategic development of elite *S. miltiorrhiza* germplasm with enhanced tanshinone biosynthesis capacity.

The *SmCPS1* encodes a rate-limiting diterpene synthase pivotal to tanshinone biosynthesis in *S. miltiorrhiza*, exhibiting root-specific expression patterns that surpass other tissues by orders of magnitude. The transcript abundance of *SmCPS1* demonstrates a strong positive correlation with tanshinone I and IIA accumulations [29]. Furthermore, the cis-regulatory analysis identified multiple conserved motifs in the *SmCPS1* promoter, including binding sites for transcription factors governing organogenesis, photo-responsive carbon allocation, and multi-stress adaptation mechanisms [10, 30].

The uORF-mediated translational control serves as a critical regulatory node for optimizing plant-specialized metabolism and pathogen defense. The successful integration of uORF engineering with CRISPR/Cas9-based genome editing underscores its transformative potential in precision crop improvement [24]. Functional validation across model and agronomic species (*Arabidopsis thaliana*, *Lactuca sativa*, *Solanum lycopersicum*, and *Oryza sativa*) has demonstrated that targeted uORF modifications enhance multigenic traits spanning developmental regulation, ROS scavenging capacity, ascorbate biosynthesis, and phenological adaptation [27, 31–33]. A recent study precisely engineered the uORFs within the promoter of the tomato tryptophan decarboxylase gene (*SlTDC1*). This modification effectively alleviated the inhibitory effect of the uORFs on the translation of the mORF, yielding novel tomato germplasm with significantly elevated serotonin levels (1.8- to 3.1-fold higher) [34].

Our work establishes that *SmCPS1* exhibits preferential root-specific expression and plays a pivotal role in modulating tanshinone biosynthesis in *S. miltiorrhiza* (Fig. S1). Bioinformatic interrogation of the *SmCPS1* 5′UTR identified 5 uORFs as translational control elements. Functional validation through transient expression in tobacco leaf epidermis demonstrated that systematic AAA replacement of 5 uORF-initiation codons in the 5′UTR-Luciferase reporter construct induced 2.3-fold translational enhancement, as quantified by dual-luciferase assays, while maintaining unaltered transcriptional activity (Fig. 1E and F). These observations mechanistically align with conserved eukaryotic translational repression mechanisms mediated by uORF architecture [21, 31]. Although the locus silencing was experimentally confirmed *in vitro*, a notable limitation of this approach became apparent. Specifically, the suppression of endogenous SmCPS1 protein translation abundance in *S. miltiorrhiza* plants could not be adequately evaluated due to the lack of target-specific antibodies. We evaluated the effect of uORF alterations on SmCPS1 protein by heterologously expressing Flag-tagged SmCPS1. Western blot analysis revealed that deletion of the uATG resulted in a marked increase in SmCPS1 protein expression levels (Fig. 2H). Identified 5 uORFs within the 5′ leader sequence of *SmCPS1*, forming a targetable regulatory reservoir distinct from conventional approaches. In contrast to promoter editing or overexpression strategies, which risk transcriptional silencing or metabolic perturbations, uORF disruption selectively enhances translational efficiency, enabling precise modulation of enzyme abundance without altering mRNA stability [35, 36]. Building on the CRISPR/Cas9-mediated multiplex editing strategy successfully deployed in rice for fine-tuning heading dates through critical flowering gene regulatory regions [37]. We aim to establish *S. miltiorrhiza* lines with graded tanshinone accumulation through differential translational regulation of *SmCPS1*. Mechanistic validation emerged from mutant lines exhibiting unchanged *SmCPS1* transcript levels coupled with elevated protein accumulation, confirming that uORF editing primarily operates at the translational tier. This finding aligns with the established uORF-mediated regulation of stress-responsive genes in *Arabidopsis* and rice, which involves adjustments in ribosome loading rates [26, 38].

Notably, the *SmCPS1* uORF mutant lines demonstrated synchronized transcriptional upregulation of some key tanshinone biosynthetic genes (*SmIPPI*, *SmHMGR1*, *SmKSL1*, *SmCYP76AH1*, *SmCYP76AH3*), implying the induction of compensatory feedback mechanisms through elevated metabolic flux (Fig. 4 and Fig. S8). While *SmCPS1* transcript levels remained unchanged, the expansion of its enzymatic product pool notably triggered coordinated activation of both upstream and downstream biosynthetic pathway genes. This phenomenon bears mechanistic parallels to feedback regulatory mechanisms observed in *Glycyrrhiza uralensis* roots, where glycyrrhizin accumulation modulates triterpenoid saponin biosynthesis through analogous pathway coordination [39]. This systemic metabolic interplay underscores *S. miltiorrhiza*'s inherent capacity to exploit precursor abundance, effectively magnifying the translational enhancement impact at individual genetic loci. However, the specific mechanisms by which uORF knockout regulates genes beyond S*mCPS1*, including structural genes and transcription factors, remain to be fully elucidated. Additionally, the precise regulatory crosstalk between these genes requires further investigation. Such network-level propagation of single-gene modifications reinforces the critical need for holistic pathway analysis in metabolic engineering paradigms [40, 41].

The uORF-knockout-mediated translational enhancement of *SmCPS1* confers unique advantages compared to traditional transgenic methodologies. The preservation of endogenous promoter architecture and transcript integrity minimizes epigenetic drift: a persistent challenge in constitutive overexpression systems [42]. Furthermore, the modular design principle permits precise dose-responsive control of protein production, extendable through multiplex uORF editing or strategic combinatorial manipulation of rate-limiting enzymatic nodes [43]. uORF editing-mediated translational control enables metabolic enhancement through optimized translation efficiency of existing transcripts without imposing a cellular burden [44]. The regulatory effects of uATG on protein levels displayed positional heterogeneity, which could be attributed to multiple factors, including flanking sequence context, RNA secondary structure, and characteristic features of the 5′UTR, such as length and GC content [45–49]. Notably, while both Del 1# and Del 4# mutants maintained identical uORF counts to CK, Del 1# demonstrated a measurable increase in TTA. This enhancement may be associated with structural modifications in the 5′UTR secondary structure induced by a nearly 29-bp nucleotide deletion upstream of the uORF initiation site. The observed superior tanshinone production in Del 2# compared to Del 3# likely stems from uORF types, differential RNA secondary architectures, variations in uORF length, and GC content disparities within their respective regulatory regions (Figs 2G, 3C–G, and Fig. S6). Regrettably, the roles of uORF-4 and uORF-5 could not be verified in this study due to the lack of stable edited plants. Future research could focus on developing targeted base editors for the uATG of each uORF, thereby enabling the functional characterization of individual uORFs. Additionally, optimizing the *S. miltiorrhiza* gene editing knockout system to enhance knockout efficiency would generate additional genome-edited materials for verifying the function of each uORF.

Our study establishes a practical precision breeding framework for *S. miltiorrhiza*, achieving up to 1.81-fold tanshinone enhancement through uORF editing while demonstrating its functional utility in medicinal plant metabolic engineering (Fig. 3G). This is highly relevant to previous research in which uORFs in the *SlTDC1* gene were precisely engineered using CRISPR/Cas9 technology, yielding novel tomato germplasm with significantly elevated serotonin levels; however, the current study extends this approach to investigate the regulatory role of uORFs in terpenoid biosynthesis in medicinal plants [34]. Subsequent field trials should evaluate translation-enhanced lines for root biomass allocation and stress resilience. Crucially, transcription factors (TFs) modulate bioactive compound accumulation through coordinated regulation at multiple biosynthetic nodes [50]. CRISPR/Cas9-mediated targeted editing has been employed to disrupt the genes encoding *bZIP1* and *bZIP2*, transcription factors acting as negative regulators of tanshinone biosynthesis. Plants harboring knockout mutations in either gene exhibited darker root coloration and elevated levels of lipophilic tanshinones. This approach parallels our strategy of using gene editing to alleviate uORF-mediated translational repression of *SmCPS1* [51]. Over recent decades, functionally characterized TFs from diverse families, including *bHLH* (basic helix–loop–helix), *ERF* (ethylene-responsive factor), and *MYB* (myeloblastosis) types, have been shown to positively mediate tanshinone biosynthesis regulation [52]. Future investigations could extend uORF editing targets to transcriptional activators of tanshinone biosynthesis, such as *SmMYB98*, thereby enabling multidimensional optimization of both metabolic production and stress adaptation in *S. miltiorrhiza* [53]. Our findings further illuminate 5′ leader sequence engineering's potential in medicinal plant synthetic biology, where the rational design of mRNA secondary structures or internal ribosome entry sites within 5′UTR may offer complementary translational control strategies. Integration with machine learning-assisted uORF regulatory strength prediction opens avenues for *de novo* design of synthetic leaders with tailored metabolic flux, establishing novel plant engineering paradigms.

This strategy proves particularly advantageous for energy-intensive enzymes like diterpene synthases, where protein synthesis constitutes the rate-limiting step. The evolutionary conservation of uORF regulatory mechanisms across species suggests broad applicability in medicinal plant engineering for terpenoid, alkaloid, and phenylpropanoid pathways. Our work raises scientific considerations in the future: (i) Systematic evaluation of multiplex uORF editing strategies to coordinately enhance rate-limiting enzymatic steps, with particular emphasis on harnessing *SmCPS1*-mediated epistatic effects for synergistic metabolic network optimization; (ii) Development of inducible or tissue-specific uORF editors could resolve yield/development trade-offs inherent to constitutive editing.

Functioning as a predictable metabolic amplifier, uORF editing bridges genetic manipulation with translational control, resolving the historical compromise between metabolite yield and plant fitness. Advanced CRISPR toolkits coupled with multi-omics integration will accelerate elite *S. miltiorrhiza* cultivar development through synthetic biology platforms, heralding a new era of precision phytochemical production.

## Materials and methods

### Plant material and chemicals

The experimental materials consisted of *S. miltiorrhiza* (purple-flowered cultivar) collected from Laiwu City, Shandong Province, China. *S. miltiorrhiza* specimens were maintained under controlled environmental conditions (24 ± 2°C) with a 16-hour photoperiod during gene-edited seedling development. Following this phase, plants were acclimatized in the greenhouse facility at Shanghai Jiao Tong University's School of Agriculture and Biology. *N. benthamiana* was grown in a 1:2 (v/v) peat soil-vermiculite mixture under controlled growth chamber conditions: 23 ± 2°C temperature regime, 16/8-hour light/dark cycle with 3000 lux illumination intensity, supplemented with regular irrigation and nutrient applications. Analytical standard reagents, including Tan IIA, CPT, DHT, and Tan I, were procured from Shanghai Yuanye Bio-Technology Co., Ltd.

### DNA and RNA extraction, real-time quantitative PCR

One-month-old gene-edited and wild-type *S. miltiorrhiza* lines were used as materials for total DNA extraction using the cetyltrimethyl ammonium bromide (CTAB) method [54]. Approximately 100 mg of tissue was ground into powder in liquid nitrogen, and total RNA was extracted using the RNApure Plant Kit (*KWBIO*, Shanghai, China) according to the manufacturer's instructions. First-strand cDNA synthesis was performed with 1 μg total RNA using Takara's PrimeScript II First Strand cDNA Synthesis Kit (Dalian, China). qRT-PCR was conducted using Takara's 2× SYBR^®^ Premix Ex Taq™ II (Tli RNaseH Plus) (Dalian, China) on a Roche LightCycler 96 real-time PCR system (Roche, Germany), with 50× diluted cDNA as template. *SmActin* and *NbActin* served as the internal reference genes, and relative quantification was calculated using the 2^−ΔΔCt^ method. The primer sequences are shown in Table S2.

### Positive transformation identification and editing type characterization

DNA was extracted from 3-week-old *S. miltiorrhiza* seedlings for positive transformation identification and editing type characterization. Specific primers were designed to precisely amplify the Cas9 gene. The presence of the Cas9 gene was confirmed by PCR amplification and sequencing, thereby determining the transformation efficiency of the CRISPR/Cas9 system in *S. miltiorrhiza* plants.

Identification primers were designed at 200 bp upstream and downstream of the target editing site, followed by PCR amplification and sequencing to confirm gene editing events. Purified PCR fragments were cloned into the pLB intermediate vector, transformed into *Escherichia coli*, and subjected to Sanger sequencing to determine specific editing types (homozygous, chimeric, or heterozygous).

### Definition of 5′UTR and uORF of the *SmCPS1* gene

The local BLASTn analysis of *SmCPS1* coding sequences against *S. miltiorrhiza* transcriptomic data identified a 192-bp 5′UTR upstream of the CDS initiation site. Within this regulatory region, a conserved uORF was characterized, flanked by a canonical initiation codon (uATG) and multiple stop codons (TAG, TGA, and TAA). The visualization analysis of uORF in this study was accomplished using the R language (version 4.3.1).

### Plasmid construction and plant transformation

The construction of gene-editing plasmids uses highly efficient elements screened through the transient transformation of *S. miltiorrhiza* protoplasts, as previously reported [55]. Our gRNA design optimization employed dual strategic approaches: (i) precise PAM site localization adjacent to uORF-associated uATG domains, coupled with (ii) integration of four unique gRNA architectures to enhance the generation of molecularly heterogeneous knockout variants. This combinatorial methodology enabled the systematic assembly of a functionally diverse mutant repository for comprehensive uORF interrogation. The high-quality *S. miltiorrhiza* genome (IMPLAD_Smil_shh, GenBank assembly: GCA_028751815.1) served as the reference for CRISPR guide RNA design using CRISPR Direct (http://crispr.dbcls.jp/), followed by a comprehensive evaluation of guide efficacy and off-target potential through CRISPOR analysis (https://crispor.gi.ucsc.edu/) [56–58]. This dual-platform computational pipeline enabled systematic selection of optimal sgRNAs with minimized off-target effects. Four sgRNAs targeting the *SmCPS1* 5′UTR, gRNA1: 5′-CCTAAACCCTGTTCCGCCAC-3′; gRNA2: 5′-TGGCAGTCAAATGAAAAATG-3′; gRNA3: 5′-TGAGCTACCGCAGACAAGCA-3′; gRNA4: 5′-CTTTCCACATCTCCACTCAA-3′, were cloned into scaffold sequences and assembled as tRNA-processed polycistronic units under the transcriptional control of the endogenous *SmU6.1* promoter. A plant-codon-optimized *Cas9* gene, driven by the *SlEF1α* promoter, was incorporated into the CRISPR vector backbone engineered from the *pCAMBIA1300* plant expression system. Tissue-cultured sterile explants were inoculated with *Agrobacterium tumefaciens* C58 harboring either the *pCAMBIA1300sm-SmCPS1-uORF* construct or the empty *pCAMBIA1300sm* control vector.

For dual-luciferase reporter vector construction, the plant expression vector *pGreenII-0800* was cleaved using restriction endonucleases *Pst*I and *Hin*dIII. Recombinant *SmCPS1* promoter and differently cloned uORFs from *S. miltiorrhiza* mutant lines were DNA into the linear vector using the One Step Cloning Kit (Vazyme Biotech). *Agrobacterium* strain GV3101 (pMP90, *pTiC58DT-DNA*) was used for tobacco transient transformation. The *Agrobacterium* strain was grown overnight in Luria-Bertani liquid medium at 28°C with 200 rpm. Centrifuge the cells at 3000 *g* for 5 minutes, resuspend in 10 mmol∙l^−1^ MES, 10 mmol∙l^−1^ MgCl_2_, 200 μmol∙l^−1^ acetosyringone, pH 5.7, and adjust to A_600_ = 0.8. The cell suspension was incubated in the dark at room temperature for 3 hours and then injected into the underside leaves of 6-week-old *N. benthamiana*. After 24 hours of dark cultivation, transfer to a greenhouse. Two days later, samples were taken for LUC (firefly luciferase)/REN (Renilla luciferase) mRNA activity and LUC/REN activity measurement.

RNA was extracted from ~100 mg of transiently transformed tobacco leaf tissue ground to a powder in liquid nitrogen, and reverse transcribed into cDNA. LUC/REN mRNA activity was determined by quantifying LUC and REN mRNA levels separately using real-time quantitative PCR. The LUC/REN mRNA activity ratio was then calculated. The *NbActin* served as the internal reference gene, and relative quantification was performed using the 2^−ΔΔCt^ method. Primer sequences are listed in Table S.

As for LUC/REN activity measurement, following the manufacturer's guidelines, utilize the dual luciferase reporter assay system to assess the functions of LUC and REN, and determine the ratio of LUC activity to REN activity. Each trial includes three separate biological replicates.

The *A. tumefaciens* EHA105 leaf disc transformation system was employed to generate transgenic *S. miltiorrhiza* plants with *SmCPS1* uORF knockout. Laboratory-maintained sterile seedlings served as negative controls. Briefly, healthy seedlings from tissue culture bottles were subjected to a 2-day dark pretreatment before 30 minutes of immersion in *A. tumefaciens* EHA105 suspension (MS medium supplemented with acetosyringone). Following co-cultivation, explants were transferred to MS regeneration medium containing appropriate antibiotics for subsequent subculturing to induce shoot formation and root development.

### Western blot analysis

The recombinant constructs containing distinct uORF variants (CK, Del 1#, Del 2#, Del 3#, and Del 4#) fused to the full-length *SmCPS1* coding sequence were independently cloned into pHB-X-Flag vectors under the driven of the native *SmCPS1* promoter. These constructs were then introduced into *A. tumefaciens* strain GV3101 (pSoup). Positive transformants were grown overnight in LB medium and adjusted to OD600 = 0.8 in liquid MS medium. Bacterial suspensions of equal volume were combined and coinfiltrated into leaves of 4-week-old *N. benthamiana* plants. Leaf discs of equal size samples were collected following 24-hour dark and 24-hour low light incubations and ground into powder in liquid nitrogen. The powder was then re-suspended in extraction lysis buffer (50 mM Tris–HCl, pH 7.4, 150 mM NaCl, 10% glycerol, 1 mM EDTA, 0.5% Nonidet P-40) supplemented with protease inhibitors (100 μM pefabloc and 100 μM cocktail). After incubating on ice for 10 minutes, samples were centrifuged at 12 000 rpm for 10 minutes. Protein quantification was performed with the Bradford Protein Assay Kit (TaKaRa).

For western blot analysis, 50 μg of total protein was loaded per sample. And the anti-Flag antibody (1:4000 dilution; Sigma-Aldrich, USA) was used to assess SmCPS1 protein accumulation levels, with the Coomassie brilliant blue-stained rubisco large subunit gel shows equivalent sample loadings. The relative intensity of the SmCPS1 protein band was normalized using ImageJ software.

### Determination of tanshinone content

Root tissues from 1-year-old *S. miltiorrhiza* plants underwent sequential processing: thorough washing, liquid nitrogen flash-freezing, vacuum lyophilization, and mechanical pulverization. Methanol (HPLC-grade) extraction was employed to isolate tanshinones for subsequent analysis. Quantitative determination of individual tanshinones (Tan IIA, CPT, DHT, and Tan I) was performed via UHPLC-TQMS (TQ-Absolute system). The operation method of UHPLC-TQMS refers to a previous study [13]. Total tanshinone accumulation (TTA) was calculated as the summation of all four quantified individual tanshinones.

## Supplementary Material

Web_Material_uhaf249

## Data Availability

The data that support the findings of this study are available in the supplementary material of the article.
