# Supplementary material for: CRISPR/Cas9-mediated uORF engineering enhances tanshinone biosynthesis in Salvia miltiorrhiza
Source: Hortic Res. 2025 Sep 17;13(1):uhaf249. doi: 10.1093/hr/uhaf249 (PMC12856501; doi:10.1093/hr/uhaf249)

Schematic diagram of the position of uORFs in the full-length sequence of *SmCPS1*

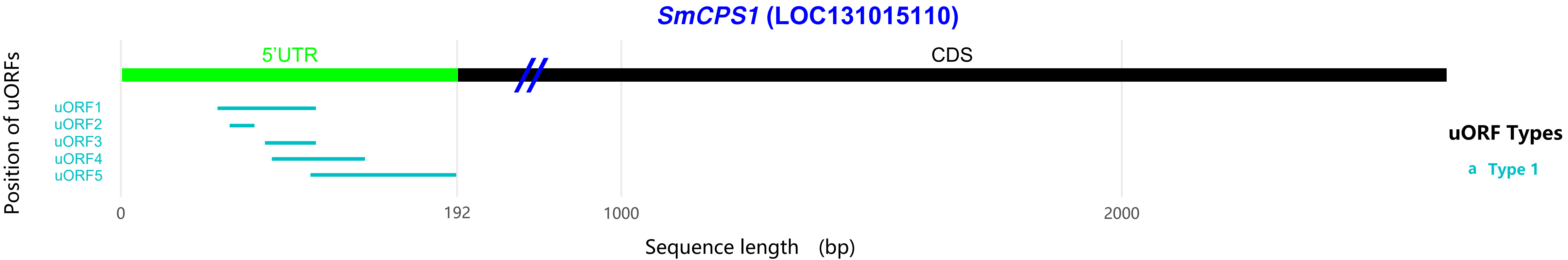

Supplement: Web_Material_uhaf249 [file web_material_uhaf249.zip › Fig. S2.pdf]
